# A Novel sRNA in *Shigella flexneri* That Regulates Tolerance and Virulence Under Hyperosmotic Pressure

**DOI:** 10.3389/fcimb.2020.00483

**Published:** 2020-09-16

**Authors:** Guang Yang, Boan Li, Leili Jia, Huaiyu Qiu, Mingjuan Yang, Binghua Zhu, Jing Xie, Shaofu Qiu, Peng Li, Hui Ma, Hongbin Song, Ligui Wang

**Affiliations:** ^1^Center for Disease Control and Prevention of Chinese People's Liberation Army, Beijing, China; ^2^The 5th Medical Center of General Hospital of Chinese People's Liberation Army, Beijing, China; ^3^Department of Ophthalmology, Beijing Chaoyang Hospital, Capital Medical University, Beijing, China; ^4^305 Hospital of PLA, Beijing, China; ^5^The 6th Medical Center of General Hospital of Chinese People's Liberation Army, Beijing, China

**Keywords:** *Shigella flexneri*, sRNA, environmental stress, virulence, *Ssr54*

## Abstract

Regulation of the environmental stress response and virulence of *Shigella flexneri* may involve multiple signaling pathways; however, these mechanisms are not well-defined. In bacteria, small regulatory RNAs (sRNAs) regulate bacterial growth, metabolism, virulence, and environmental stress response. Therefore, identifying novel functional sRNAs in *S. flexneri* could help elucidate pathogenic adaptations to host micro-environmental stresses and associated virulence. The aim of this study was to confirm the presence of an sRNA, *Ssr54*, in *S. flexneri* and to determine its functions and possible mechanism of action. *Ssr54* was found to regulate tolerance and virulence under hyperosmotic pressure. Its expression was verified by qRT-PCR and Northern blotting, and its genomic position was confirmed by 5′-rapid amplification of cDNA ends. *Ssr54* expression was significantly decreased (~ 80%) under hyperosmotic conditions (680 mM NaCl), and the survival rate of the *Ssr54* deletion strain increased by 20% under these conditions. This suggested that *Ssr54* has been selected to promote host survival under hyperosmotic conditions. Additionally, virulence assessment, including guinea pig Sereny test and competitive invasion assays in mouse lungs, revealed that *Ssr54* deletion significantly decreased *S. flexneri* virulence. Two-dimensional gel analyses suggest that *Ssr54* may modulate the expression of *tolC, ompA*, and *treF* genes, which may affect the virulence and survival of *S. flexneri* under osmotic pressures. Furthermore, *treF* expression has been shown to improve the survival of *S. flexneri* under osmotic pressures. These results suggest that *Ssr54* has a broad range of action in *S. flexneri* response to hyperosmotic environmental stresses and in controlling its virulence to adapt to environmental stresses encountered during host infection.

## Introduction

*Shigella* is a genus of gram-negative enteric bacteria, accounting for some of the most prominent pathogens causing bacterial diarrhea via fecal-oral route. *Shigella* infect colonic epithelial cells, leading to typical bacterial diarrheal symptoms. *S. flexneri* is the most prevalent endemic strain in developing countries (Kotloff et al., 1999; Wang et al., 2006). When *S. flexneri* enters the host body, it faces various host gastrointestinal environmental stresses characterized by fluctuating temperature, nutrients, bile salts, and pH conditions. Adaptations to these environmental stresses are important for *S. flexneri* survival and subsequent invasion and colonization of the intestinal tract, which are responsible for the pathogenic effects of the infection. To adapt, the bacteria require coordinated regulation of gene expression for rapid responses to and tolerance of these stresses. For instance, in *S. flexneri*, acid resistance is dependent on pH and controlled by RpoS under stress conditions (Small et al., 1994). Meanwhile, the two efflux pumps, AcrAB and MdtJI, of *Shigella* are reportedly associated with resistance to bile salts and the extrusion of toxic compounds (Leuzzi et al., 2015; Nickerson et al., 2017). Hence, developing strategies for tolerating changes in environmental osmolarity, is critical for pathogenic bacteria to not only permit survival during environmental osmotic stress, but also to establish infection (Mahmoud et al., 2017). Specifically, maintaining a stable osmotic balance between the cell cytoplasm and outer environment is an important challenge for all cell types, particularly bacteria.

Different bacteria respond to environmental stresses via different strategies, including the use of small regulatory RNAs (sRNAs). Most sRNAs are located in intergenic regions that range from 40 to 500 nt. They affect mRNA activity by base pairin with the 5'-untranslated regions (5′-UTRs) and/or the ribosome binding site (RBS) of these targets (Gottesman and Storz, 2011; Vanderpool et al., 2011). Antisense sRNAs, transcribed on the DNA strand opposite their target gene, are one type of sRNAs that act via extensive base pairing (Wagner and Romby, 2015). sRNAs have been identified in many bacteria, including *Escherichia coli, Staphylococcus aureus*, and *Vibrio cholerae*, in which they play pivotal roles in bacterial adaptation to environmental stress and virulence (Lenz et al., 2004; Mangold et al., 2004; Pichon and Felden, 2005; Bobrovskyy and Vanderpool, 2013; De Lay et al., 2013; Murina and Nikulin, 2015). For example, *E. coli* DsrA can bind to RpoS mRNA, which encodes the alternative sigma factor S, to repress formation of a closed secondary structure in the RBS of *rpos* transcripts and promote translation (Massé and Gottesman, 2002). Moreover, *E. coli* sRNA *RyhB* regulates intracellular Fe^2+^ utilization and storage, as well as iron binding proteins (Massé et al., 2005). sRNA also affects the pathogenesis of gram-positive *Enterococcus faecalis* by regulating its growth and survival under various environmental stresses, including iron stress (Michaux et al., 2014). In fact, studies have suggested that ~10–30% of all bacterial genes are subjected to sRNA regulation (Lewis et al., 2005).

Various studies have also reported on sRNA function in *Shigella*. For instance, in *S. dysenteriae*, the sRNA *RyfA1* negatively impacts the virulence-associated process of cell-to-cell transfer, as well as expression of *ompC*, a gene that is important for the pathogenesis of *Shigella* (Fris et al., 2017). Additionally, CsrB/CsrC is regulated in response to carbon availability, RyhB is regulated in response to iron availability, while RnaG, as well as the activity of the shuA RNA thermometer, is regulated in response to temperature (Fris and Murphy, 2016). RnaG (450 nt), a small non-coding RNA encoded on the virulence plasmid pINV of *Shigella flexneri*, is able to directly interact with *icsA* mRNA, thereby promoting premature termination of transcription (Giangrossi et al., 2010) Additionally, VirF, which generally assists the RNA–RNA interaction, has been shown to directly activate the transcription of the small non-coding RnaG and *icsA* mRNA, which encodes two essential proteins that are involved in the pathogenicity process by binding their promoter regions (Giangrossi et al., 2017). *RyhB* (90 nt) modulates and inactivates ferritin-related genes to regulate iron metabolism and can inhibit the expression of virulence genes such as *virB*, a regulator of the T3SS secretory protein gene (Murphy and Payne, 2007). Meanwhile, in our previous study, *Ssr1* (150 nt) was shown to help bacteria adapt to an acidic environment by regulating acid resistance genes and to regulate bacterial virulence by controlling the expression of T3SS genes (Wang L. et al., 2016). However, the functions of many other predicted sRNAs in *Shigella* have not yet been elucidated. The identification of novel *S. flexneri* sRNAs is pivotal to understanding *Shigella* virulence and adaptation to host microenvironment. Therefore, the aim of the present study was to confirm the presence of an sRNA, *Ssr54*, in *S. flexneri* that was predicted using a comparative genomics bioinformatics screening method (Wang L. et al., 2016), as well as determine the functions and possible mechanisms of action of this sRNA in *S. flexneri*.

## Materials and Methods

### Bacterial Strains, Growth Media, and Stress Conditions

The wild-type strain used in this study was *S. flexneri* 2a 301. This strain was cultured on Congo red solid culture medium at 37°C in 5% CO_2_ overnight. A single colony was selected, inoculated in liquid Luria-Bertani (LB) medium, and incubated at 37°C on an orbital shaker at 150 rpm for 6–8 h. When necessary, kanamycin, ampicillin, or chloramphenicol was added to the culture medium to obtain a final concentration of 50, 100, or 30 μg/mL, respectively. In the stress test, a bacterial culture with an OD_600_ of 0.6 was pelleted, resuspended in PBS, and inoculated into the following culture media: a hypoosmotic LB culture medium not containing NaCl, hyperosmotic LB culture media containing 340 or 680 mM NaCl, and LB culture medium with 160 mM NaCl as the negative control. The bacteria were cultured under these conditions for 30 min. Thereafter, we assessed sRNA expression by qRT-PCR and performed the survival assay under hyperosmotic conditions.

For the northern blot analysis, wild-type, *Ssr54* mutant, and *Ssr54* complement strain cells were harvested during the logarithmic growth phase (OD_600_ = 0.6). When required, the cultures were subjected to hypo/hyperosmotic stress conditions as described above. Non-stressed exponentially growing cultures were used as controls.

### RNA Extraction and RT-PCR

Total RNA was isolated with TRIzol (Invitrogen, Carlsbad, CA, USA, 15596108) from S. *flexneri* cultured to the middle logarithmic growth phase (OD_600_ = 0.6), and remaining DNA was digested with DNase (Promega, Madison, WI, USA, M6101). ODs were determined at 260 nm and 280 nm using a UV-Vis spectrophotometer to assess RNA concentration. DNase-treated total RNA (not more than 10 μg) was reverse-transcribed into cDNA using a reverse transcription kit (Promega, Madison, WI, USA, K1005S) in accordance with the manufacturer's instructions. cDNA was synthesized using 2 μg of total RNA and a Promega Reverse Transcription System. It was then diluted (1:5) in nuclease-free water. Primers were designed based on the *Ssr54* sequence (Supplementary Table 1 for further details) and used the cDNA as PCR template. PCR was performed using ExTaq Mix (Takara Biochemicals, Beijing, China, RR001Q) under the following thermal cycling conditions: 10 min at 95°C for pre-incubation, followed by 30 cycles of amplification (30 s at 95°C, 30 s at 55°C, and 30 s at 72°C) (Absin Bioscience, Foster City, CA, USA). RNA preparation and qRT-PCR analysis were repeated at least three times.

### Northern Blot

*Ssr54* size and expression levels were assessed by northern blotting with total RNA from the wild-type, *Ssr54* mutant, and complement strains in normal and hyperosmotic conditions. Total RNA (15–20 μg from each strain) was separated via denaturing polyacrylamide gel electrophoresis (PAGE) (10% resolving gel) and electroblotted onto a nylon membrane where it was cross-linked under a 1,200-mJ UV light. The *Ssr54* probe sequence was 5′-ATTCTTCAGAGATCACAAACTGGTTA-3′. The sequence of 5S rRNA probe, which was used as the internal control, was 5′-GTTTCACTTCTGAGTTCGGCATGGGGTCAGGTGGG-3′. These probes were synthesized by Shanghai Invitrogen Life Technology Service (Shanghai, China). The RNA was then hybridized with a biotin-labeled *Ssr54* probe overnight, and the hybridization signal was detected using a North2South® Chemiluminescent Hybridization and Detection Kit from Thermo Fisher Scientific (Carlsbad, CA, USA, 17097) with an exposure time of 1–5 min.

### Mapping of sRNA by Rapid Amplification of cDNA Ends (RACE)

The precise size of *Ssr54* was determined by RACE using the 5′-Full RACE Kit (Takara Biochemicals, Beijing, China, D315) in accordance with the manufacturer's instructions. Total RNA was isolated from *S. flexneri* 2a 301 during the exponential phase (OD = 0.6). The *Ssr54* RACE outer primer and inner primer sequences are presented in Supplementary Table 1. The resulting purified sRNA-derived PCR product was cloned into the pMD18T vector and transformed into competent *E. coli* DH5α cells. The inserts were sequenced to identify the TSSs of the individual sRNAs.

### qRT-PCR

qRT-PCR was performed to assess *Ssr54* sRNA expression in *S. flexneri* under different osmotic pressures. cDNA was synthesized using 2 μg of total RNA and a Promega Reverse Transcription System and then diluted (1:5) in nuclease-free water. With 3 μL of cDNA as the template and 16S rRNA as the internal control, *Ssr54* was amplified by PCR using the 2 × SYBR Green I Master Mix protocol (Invitrogen, Carlsbad, CA, USA). The primers are presented in Supplementary Table 1. RT-PCR was performed using the ABI 7500 system (Applied Biosystems) with the following cycling conditions: 5 min denaturation step at 42°C, followed by 40 amplification cycles of 30 s at 95°C, 30 s at 55°C, and 30 s at 72°C. *Ssr54* gene transcript levels under various stress conditions were compared with those of the wild-type strain grown in normal LB medium at 37°C. Relative expression levels were calculated using the 2^−Δ*ΔCT*^ method (Gong et al., 2011). All experiments were conducted in triplicate.

### Construction of sRNA Mutant and Complement Strains

The *Ssr54* mutant strains were generated through λ-Red homologous recombination (Park et al., 2001). Genome segments 500–600 bp upstream and downstream of the homologous arm fragments in the *Ssr54* sRNA sequence were amplified. The kanamycin resistance gene from the pUC19-kan plasmid was amplified, and the fusion-PCR method (with three fragments at a ratio of 1:1:1) was employed to generate a linear target DNA fragment. This linear fragment was transformed into a *S. flexneri* strain containing the pKD46 plasmid, which encoded the λ-Red recombination system, and the strain was cultured overnight on an antibiotic selection plate containing ampicillin and kanamycin to screen for the *Ssr54-*deficient mutant strain. This strain was confirmed using primers listed in Supplementary Table 1.

To construct the complementing plasmid p*Ssr54*, DNA 200 bp upstream of the *Ssr54*, transcription start site (TSS), and its coding region was amplified from *S. flexneri* 2a 301. These primers targeted unique restriction enzyme sites, *Xba*I and *Sph*I, such that when the PCR product was digested with these restriction enzymes, it could be ligated into a similarly digested plasmid, recombination pACYC184 (Cm^R^Tc^R^), in an orientation-specific manner. Recombinant DNA products were verified by sequencing. The resulting plasmid was used to transform the *Ssr54* mutant strain via electroporation, followed by selection using chloromycetin. The final complement strain was dubbed Δ*Ssr54* + p*Ssr54*. pACYC184 was electroporated into these strains to generate vector-only (no sRNA) controls.

### Growth Analysis of *S. flexneri*

Overnight cultures grown in LB medium or LB medium containing kanamycin were diluted 1:100 in LB medium with the hyperosmotic supplement and then grown at 37°C with shaking at 200 rpm/min. At time points of 0, 2, 4, 6, 8, 10, 12, 14, 16, 18, 20, 22, and 24 h after inoculation, bacterial growth was monitored by measuring the OD_600_. Each sample was analyzed in triplicate and the assays were repeated at least three times. The average OD_600_ values were used to generate growth curves.

### Determination of Survival Rates

The wild-type and *Ssr54* mutant strains were cultured at 37°C with agitation at 160 rpm until the early logarithmic phase of growth (OD_600_ of 0.6). Bacterial cultures were centrifuged for 5 min at 4°C; then, pellets were washed three times in PBS, resuspended in hyperosmotic LB culture medium containing 680 mM NaCl, and cultured for 30 min at 37°C (Gong et al., 2011). Final bacterial cultures were serially diluted and plated onto LB plates for overnight incubation to determine viability. The number of colony-forming units per milliliter (CFU/mL) was then calculated for each of the conditions, with each bacterial strain cultured in LB culture medium at pH 7.0 (containing 160 mM NaCl) serving as the control. Results are expressed as mean percentage ± SD from experiments performed in triplicate.

### Induction of Keratoconjunctivitis in Guinea Pigs

Analysis of keratoconjunctivitis induced in guinea pigs is a standard method to assess *S. flexneri* virulence, and it allows for direct evaluation of *S. flexneri* pathogenesis (Sereny, 1955). To verify the effect of *Ssr54* sRNA on *S. flexneri* virulence, we investigated the effect of the wild-type, *Ssr54* mutant, and complement strains on the corneas of guinea pigs. Single colonies of the wild-type, *Ssr54* mutant, and *Ssr54* complement strains were selected from TSA Congo red plates and inoculated in LB culture medium containing the appropriate antibiotics for incubation at 37°C overnight. Thereafter, 1% of each culture was inoculated into 5 mL of LB culture medium and incubated until it reached an OD_600_ of 1.0 (10^6^ CFU/mL). After centrifugation for 5 min, we harvested 1 mL of each bacterial culture and resuspended it in 20 μL of PBS, which was then inoculated into the corneas of healthy guinea pigs with normal cornea and conjunctiva. Inoculated corneas were observed 24, 48, and 72 h after inoculation. Corneas inoculated with PBS served as controls. The degree of keratoconjunctivitis was graded as follows: – no keratoconjunctivitis; + mild conjunctival congestion; ++ palpebral edema, conjunctival congestion, corneal opacification, and no purulent discharge; +++ palpebral edema, corneal opacification, conjunctival congestion, and purulent discharge; ++++ palpebral edema, corneal gray patch, conjunctival congestion, and purulent discharge. The experiment was conducted in triplicate. Each group contained six guinea pigs.

### Assessment of Mouse Lung Invasion

The wild-type, mutant *Ssr54*, and complement strains were grown to the logarithmic growth phase, and then the concentrations of cultures were adjusted to 10^8^ CFU/mL. Three aliquots (1 mL each) containing the same number of bacteria were pelleted by centrifugation for 5 min at 4°C and resuspended in 1 mL of PBS. Equal amounts of the wild-type and mutant strains were mixed, and an aliquot (20 μL) of the resulting mixture (2 × 10^6^ CFU/mL), along with a similar aliquot of the mixture of equal amounts of wild-type and complement strains, were placed in the nasal cavities of BALB/c mice, under diethyl ether sedation, to infect lung tissues. After 24 h, the mice were euthanized via cervical dislocation, and lungs were collected from all and washed in RPMI (Roswell Park Memorial Institute) 1640 with 50 μg/mL gentamicin. Mouse lungs were harvested and incubated in PBS containing 0.1% Triton X-100 to release the bacteria. The extracts were then spread on either antibiotic-free or kanamycin antibiotic-containing LB plates for overnight cultivation at 37°C, and then the numbers of colonies were determined. The competitive index (CI) was defined as the ratio of mutant strain CFU after invasion to that of the wild-type strain CFU after invasion, divided by the ratio of the mutant strain CFU before invasion to that of the wild-type strain CFU before invasion. This experiment was performed in triplicate.

### HeLa Cell Invasion Assays

To further verify the influence of *Ssr54* on virulence in *S. flexneri*, a competitive invasion assay was performed using HeLa cells. *S. flexneri* strain 301, *Ssr54* mutant and complement strains were grown in LB medium to the exponential phase (OD_600_ of 0.6), adjusted to the same concentration, and mixed at a 1:1 (v/v) ratio. The two strains were added to DMEM cell culture medium (Gibco, Carlsbad, CA, USA), and the cultures formed were then added to HeLa cells at a multiplicity of infection of 100:1. After incubation for 4 h, infected HeLa cells were washed thrice with PBS and thrice with cell culture medium, following which cell culture medium containing 50 μg/mL gentamicin was added (Wang Y. et al., 2011). Gentamicin kills extracellular bacteria, while intracellular bacteria are protected from it. Following incubation at 37°C for 24 h, cell cultures were washed with PBS; the cultures were then harvested and lysed, and their cellular contents were cultured on LB and LB plates containing kanamycin. The CI was calculated, which represents the ratio of the CFUs of the recovered *Ssr54* mutant and the wild-type, complement strains and the wild-type (normalized for the input ratio), and Student's *t*-test was used to determine the *p*-value (Christiansen et al., 2004). This assay was repeated three times, and the mean was reported as the final result.

### Prediction of sRNA Targets

Proteins from the wild-type and *Ssr54* mutant strains were extracted and purified. The protein samples (total of 120 μg, as assessed by silver staining) were subjected to one-dimensional isoelectric focusing using IPG immobiline dry strip gels at pH 4–7 for 30 min at 300 V, and then 30 min at 700 V, 1.5 h at 1,500 V, and 8 h at 9,000 V until the voltage-time product reached 64 kVh. Thereafter, isoelectric focusing was discontinued, and the samples were separated in the other direction by sodium dodecyl sulfate (SDS)-PAGE (12.5% resolving gel). The gels were then stained, scanned, and subjected to image analysis. The samples underwent protein mass spectroscopy pre-treatment processes, including determination of restriction enzyme digestion sites, enzymatic digestion, peptide extraction, identification of target proteins, and mass spectroscopy measurement.

### Ethics Statement

The animals were obtained from the Laboratory Animal Center (Academy of Military Medical Sciences). All methods were carried out in accordance with the approved guidelines of the Academy of Military Medical Sciences. The experimental protocols were approved by the Ethics Committee for Animal Experimentation of the Academy of Military Medical Sciences.

### Biosecurity Statement

All standard biosecurity and institutional safety procedures were adhered to in all experimental procedures presented in this article.

## Results

### Identification of a Novel sRNA

**Figure 1:**
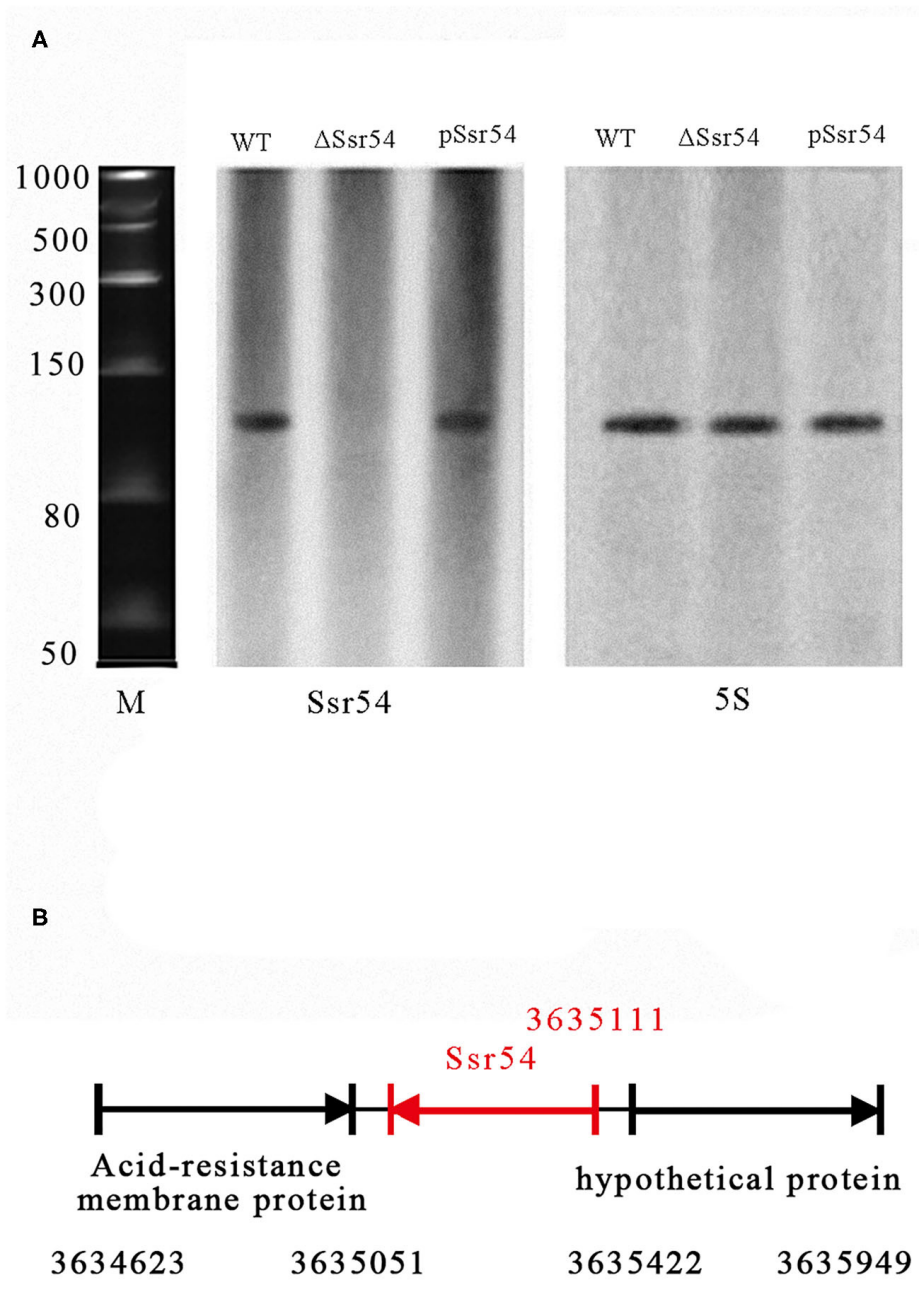
Experimental verification and expression of *S. flexneri* small RNAs (sRNAs). **(A)** Northern blot of the novel sRNA (*Ssr54*) and 5S rRNA in *S. flexneri* wild-type, *Ssr54* mutant, and *Ssr54* complement strains. *Ssr54* expression in the *S. flexneri* strains grown to logarithmic growth phase was verified by Northern blotting; **(B)** *Ssr54* genomic position in *S. flexneri* was assessed via 5′ rapid amplification of cDNA ends.

In our previous study, we predicted 57 sRNAs using a transcription unit-based method and confirmed four of these sRNAs (*Ssr1, Ssr9, Ssr44*, and *Ssr54*) by reverse transcription (RT)-PCR and northern blotting assays (Wang L. et al., 2016). In the current study, we focused on determining the characteristics and functions of *Ssr54*. sRNA *Ssr54* expression was detected by RT-PCR and verified by northern blotting. A biotin-labeled *Ssr54* probe was hybridized with a band of ~100 bp in a wild-type *S. flexneri* strain and *Ssr54* complement strain; no band was seen in the *Ssr54* deletion strain (Figure 1A). In this experiment, 5S rRNA was used as the positive control. By 5′-RACE, the *Ssr54* TSS was at 3635111 and located in a non-coding intergenic region (Figure 1B).

### *Ssr54* Regulates Osmolarity Tolerance

**Figure 2:**
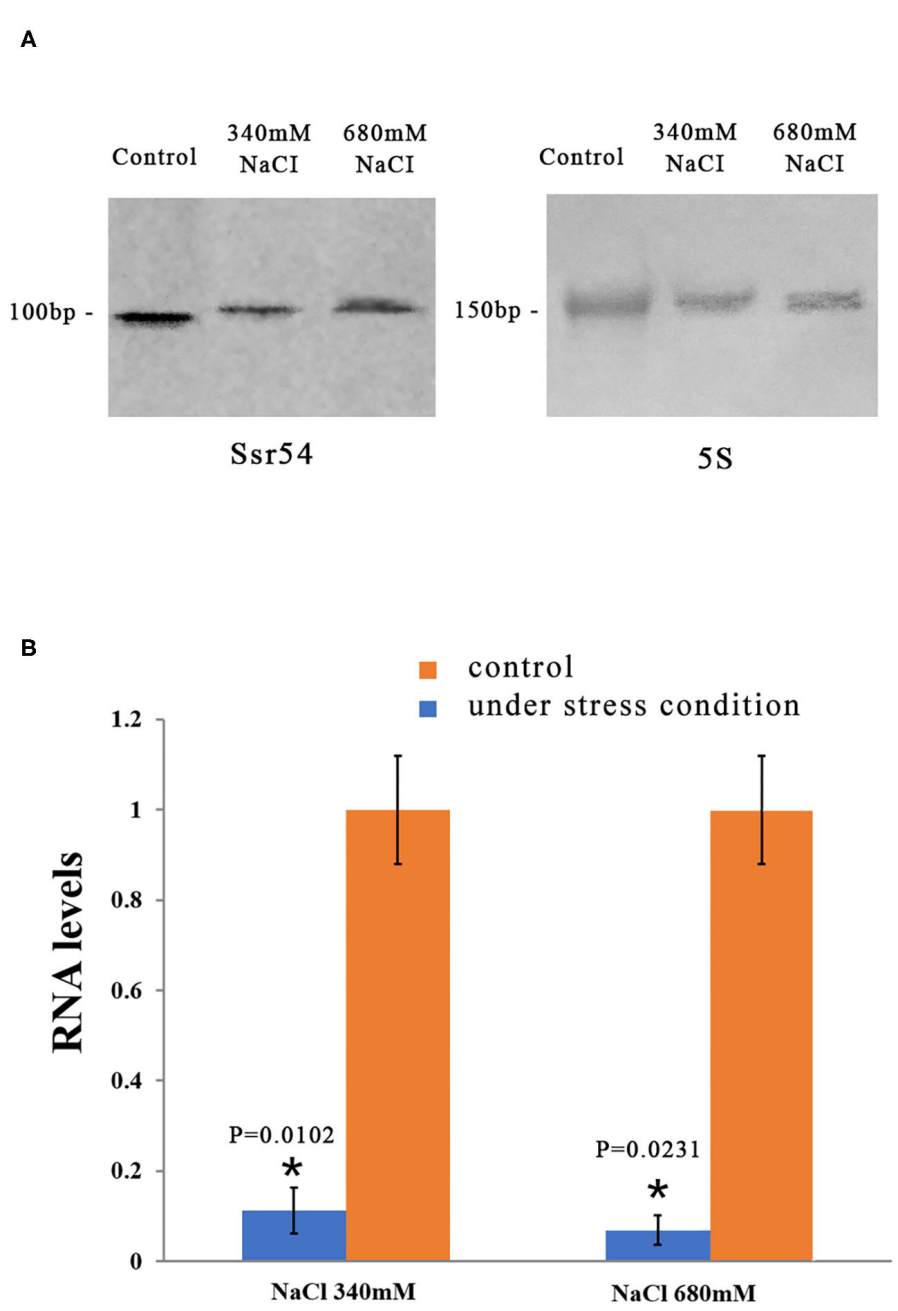
Levels of the small RNA *Ssr54* in *S. flexneri* strain 301 when exposed to high osmotic conditions for 30 min. **(A)** Northern blot of *Ssr54* and 5S rRNA in the *S. flexneri* wild-type strain under hyperosmotic conditions. Control cultured in 160 mM NaCl; **(B)** RNA levels of *Ssr54*, qRT-PCR analyses of the relative *Ssr54* levels in *S. flexneri* under high osmotic conditions compared with that in 160 mM NaCl. Relative abundance was calculated using the 2^−ΔΔCt^ method. Bars indicate fold changes calculated as the mean of triplicate experiments, representing the ratios of *Ssr54* expression levels. Black bars represent the control transcript values; Red bars represent results under stress conditions. Error bars indicate standard deviations. Statistical analyses were performed using Student's *t*-test.

We then examined whether *Ssr54* is required for *S. flexneri* to cope with osmotic stress. To this end, we first quantified its expression in osmotic conditions. Northern blot analysis was used to examine the levels of *Ssr54* sRNA in *S. flexneri* grown under various hyperosmotic conditions supplemented with 340 mM and 680 mM NaCl (Figure 2A). At osmotic pressures of 340 mM NaCl and 680 mM NaCl, the expression of *Ssr54* was predominantly low in the wild-type strain (Figure 2A). In this experiment, 5S rRNA was used as the positive control.

Reverse transcription quantitative PCR (RT-qPCR) was performed to further assess *Ssr54* expression in *S. flexneri* under different osmotic pressures. *Ssr54* was significantly downregulated under hyperosmotic pressures of 680 mM NaCl (>90% downregulation, *P* = 0.0231) and 340 mM NaCl (>80% downregulation, *P* = 0.0102) (Figure 2B). These results were consistent with the northern blot results, confirming changes in *Ssr54* expression under different osmotic pressures.

**Figure 3:**
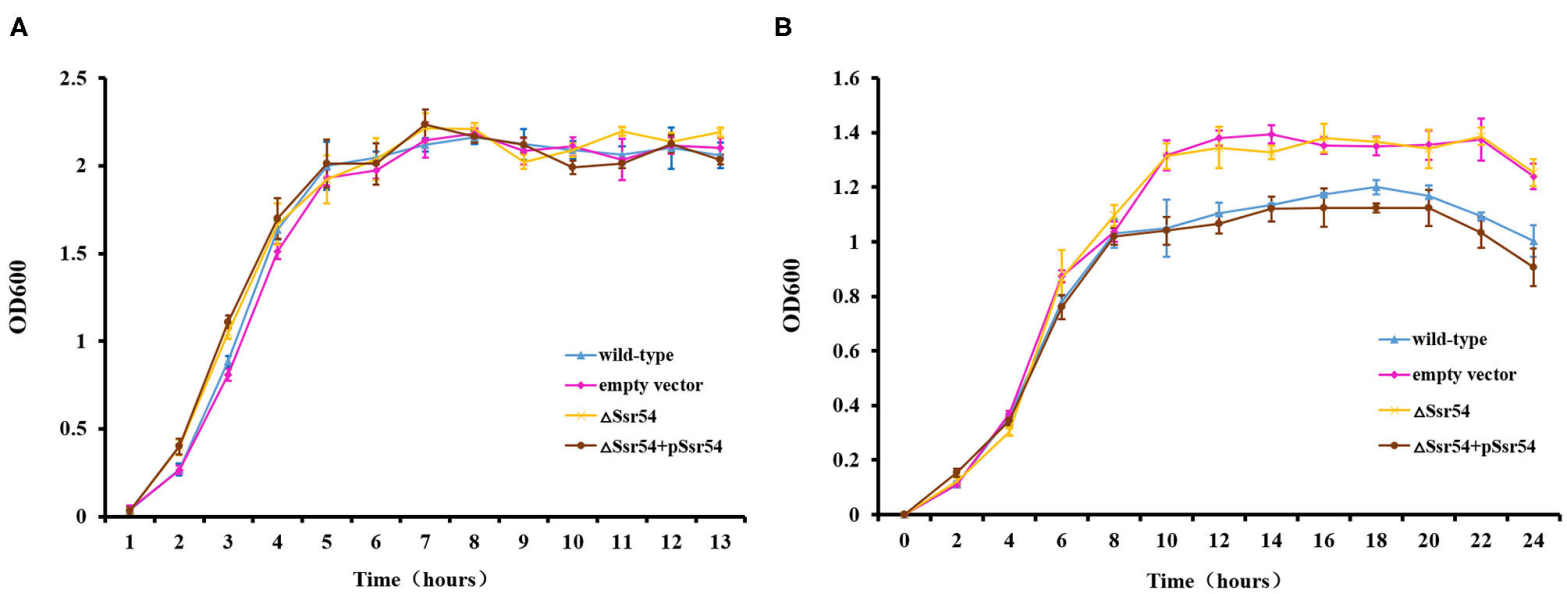
Osmotic stress of the *Ssr54* mutant. Growth characteristics of the wild-type, *Ssr54* mutant, and complement strains, as well as the mutant strain carrying the pACYC184 empty vector, in LB under different osmotic conditions: **(A)** 0 mM NaCl; **(B)** 680 mM NaCl. The error bars indicate standard deviations based on triplicate experiments. Statistical analyses were performed using Student's *t*-test.

To investigate the role of *Ssr54* in adaptation of *S. flexneri* to hyperosmotic pressures, we constructed an *Ssr54* mutant strain using λ-red homologous recombination and a low-copy plasmid *Ssr54* complement strain. All mutant and complementary *Ssr54* constructs were confirmed by PCR and agarose gel electrophoresis (Supplementary Figure 1). To determine whether *Ssr54* plays a role in regulating growth under hyperosmotic conditions, we placed the wild-type, *Ssr54* mutant, and complement strains in LB culture medium under different stress conditions at 37°C for 24 h. Optical densities at 600 nm (OD_600_) were measured every 2 h until the late stationary phase (24 h), and growth curves were plotted. A mutant carrying an empty vector served as a negative control. Under hypoosmotic conditions (0 mM NaCl), growth of the wild-type strain was not significantly different from that of the *Ssr54* mutant and complement strains (Figure 3A). Under hyperosmotic conditions (680 mM NaCl), the OD_600_ values for the *Ssr54* mutant strain were significantly higher than those for the wild-type and *Ssr54* complement strains, and the *Ssr54* mutant strain entered the stationary phase later than the wild-type, hence indicating that entry into the stationary phase was accelerated in the deletion strain (Figure 3B). This suggested that *Ssr54* expression plays an important role in *S. flexneri* growth under hyperosmotic conditions.

**Figure 4:**
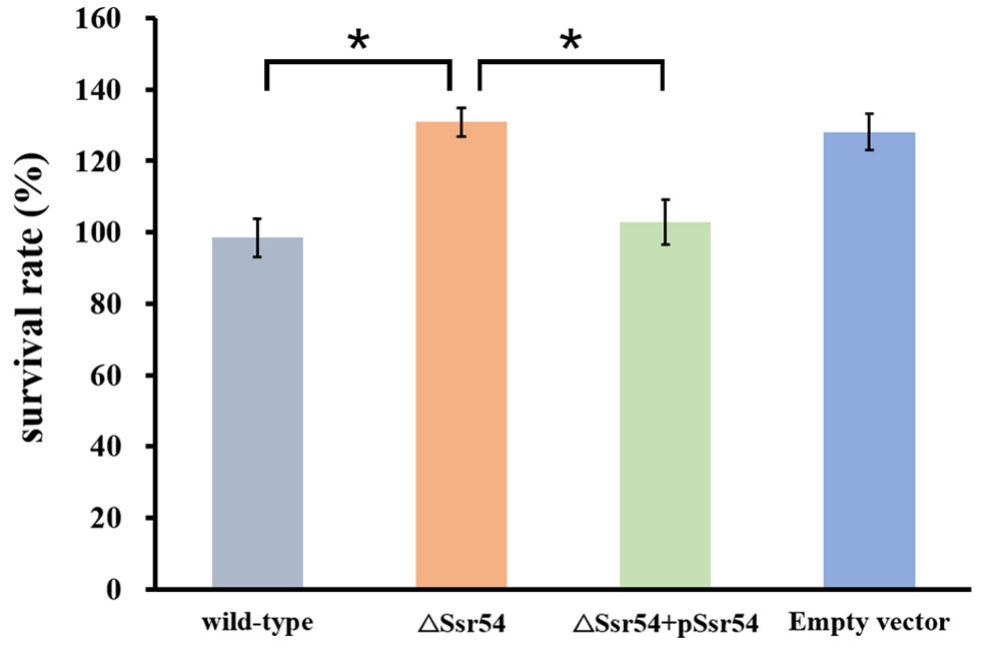
Survival of the sRNA *Ssr54* mutant strain (Δ*Ssr54*) relative to that of the wild-type *S. flexneri* 301 strain when exposed to high osmotic stress. Wild-type *S. flexneri* 301, Δ*Ssr54*, the complement (Δ*Ssr54* + p*Ssr54*) strain, and mutant strain carrying the pACYC184 empty vector were grown in Luria-Bertani medium (160 mM NaCl) up to the exponential phase and then subjected to high osmotic stress (680 mM NaCl). Viability was determined by counting bacteria plated in serial dilutions. Bars represent the mean percent survival compared with untreated controls. Statistical analyses were performed using Student's *t*-test. Data represent the average of biological triplicates, and errors bars indicate one standard deviation. *Denotes a significant difference with *P* < 0.01.

In addition, we assessed survival of the wild-type and *Ssr54* mutant strains for 30 min following hyperosmotic stimulation and found no significant difference in growth. This survival assay was used to verify survival of the wild-type and Ssr54 mutant strains under different osmolarities. After exposure to hyperosmotic pressure, survival of the *Ssr54* mutant strain improved by ~20% compared with that of the wild-type strain (*P* = 0.0109) (Figure 4). There was no difference in survival between the *Ssr54* complement strain and wild-type strain with the empty plasmid (*P* = 0.0701), indicating *Ssr54* deletion enhanced *S. flexneri* tolerance to hyperosmotic pressure and survival. The empty vector mutant served as a negative control.

### *Ssr54* Regulates Virulence

**Figure 5:**
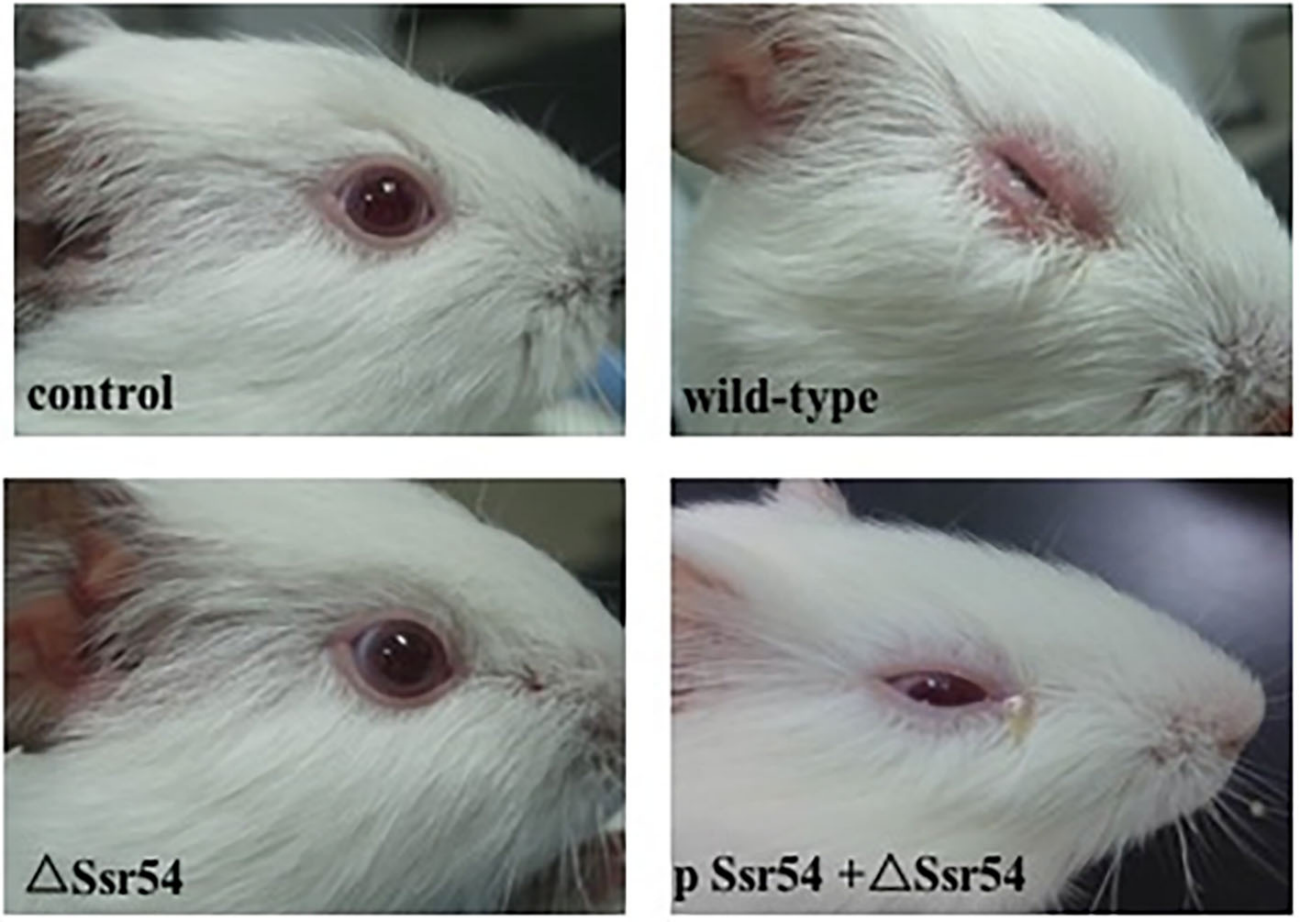
Phenotypes of wild-type *S. flexneri* and sRNA mutant Δ*Ssr54* in guinea pigs. More inflammatory cells were present in the corneas of guinea pigs inoculated with the wild-type and complement strains than those inoculated with the mutant Δ*Ssr54* strain, whereas the NaCl (control) group showed no inflammatory cells.

The wild-type, *Ssr54* mutant, and complement strains were inoculated into the corneas of healthy guinea pigs (Figure 5). After 24 h of infection, mild conjunctival congestion was observed in the wild-type and complement strain-inoculated group, whereas no keratoconjunctivitis was detected in the *Ssr54* mutant strain-inoculated group or the PBS (control) group. After 48 h of infection, the wild-type and complement strain-inoculated group presented palpebral edema and corneal opacification, and keratoconjunctivitis was still absent in the *Ssr54* mutant strain-inoculated and PBS (control) groups. After 72 h of infection, purulent discharge was observed in the wild-type and complement strain-inoculated group, and keratoconjunctivitis still remained absent in the *Ssr54* mutant strain-inoculated and PBS (control) groups (Table 1). These data indicate that there was significant inflammatory cell infiltration following inoculation with the wild-type strain; however, there was no inflammatory response following inoculation with the *Ssr54* mutant strain or PBS (Supplementary Figure 3). These data, which show the induction of keratoconjunctivitis in guinea pigs, strongly suggest that the inflammatory response time and infectivity of the *Ssr54* mutant strain were significantly less than those of the wild-type and complement strains and imply that deletion of *Ssr54* attenuates *S. flexneri* invasiveness.

**Table 1:** Keratoconjunctivitis in guinea pigs inoculated with wild-type and Δ*Ssr54 Shigella flexneri* strains or an NaCl control.

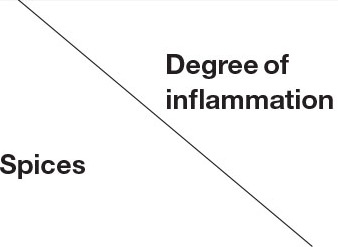	**Time**		
	**24 h**	**48 h**	**72 h**
	**The group of guinea pigs (6)**	**The group of guinea pigs (6)**	**The group of guinea pigs (6)**
Control	—	—	—
Wild-type	+	++	+++/++++
Δ*Ssr54*	—	—	—
p*Ssr54*	+	++	+++/++++

*A Sereny test showed the virulence of the sf301, ΔSsr54, and p Ssr54 strains. Guinea pig keratoconjunctivitis was rated as follows: – normal eye indistinguishable from the contralateral uninoculated eye, + lacrimation or eyelid edema, ++ lacrimation or eyelid edema plus mild conjunctival hyperemia, +++ lacrimation or eyelid edema with mild conjunctival hyperemia plus slight exudates, and ++++ full-blown purulent keratoconjunctivitis*.

**Figure 6:**
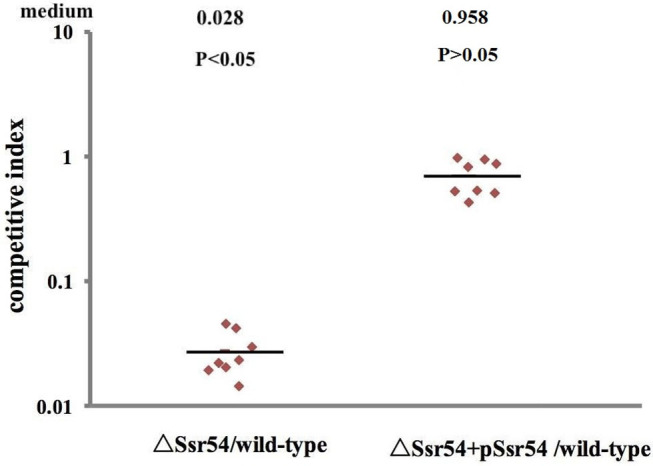
Mit4ce were infected with wild-type, mutant Δ*Ssr54*, and complement strains. A competitive index (CI) of 1 represents recovery of equivalent amounts of wild-type and mutant bacteria. The CI of the *Ssr54* mutant and wild-type was lower than 1 (*P* < 0.05); The CI of the *Ssr54* complement strain and wild-type was ~ 1 (*P* > 0.05).

Virulence of the wild-type, *Ssr54* mutant, and complement strains was also assessed using a competitive invasion model in mouse lungs. Following competitive lung invasion by the wild-type, *Ssr54* mutant, and complement strains, total viability of the *Ssr54* mutant strain (2.07 × 10^2^) was found to be significantly lower than that of the wild-type strain (6.15 × 10^4^) (*P* = 0.0068), whereas total viability of the complement strain (5.86 × 10^4^) (*P* > 0.05) was not significantly different from that of the wild-type strain. The CI between the *Ssr54* mutant and wild-type strains was 0.028 (*P* < 0.05), which indicates a significant difference, whereas that between the *Ssr54* complement and wild-type strains was 0.958 (*P* > 0.05), which was not significantly different (Figure 6). This finding suggested that deletion of *Ssr54* significantly attenuated *S. flexneri* virulence, which was consistent with the experimental data regarding keratoconjunctivitis induction in guinea pigs.

**Figure 7:**
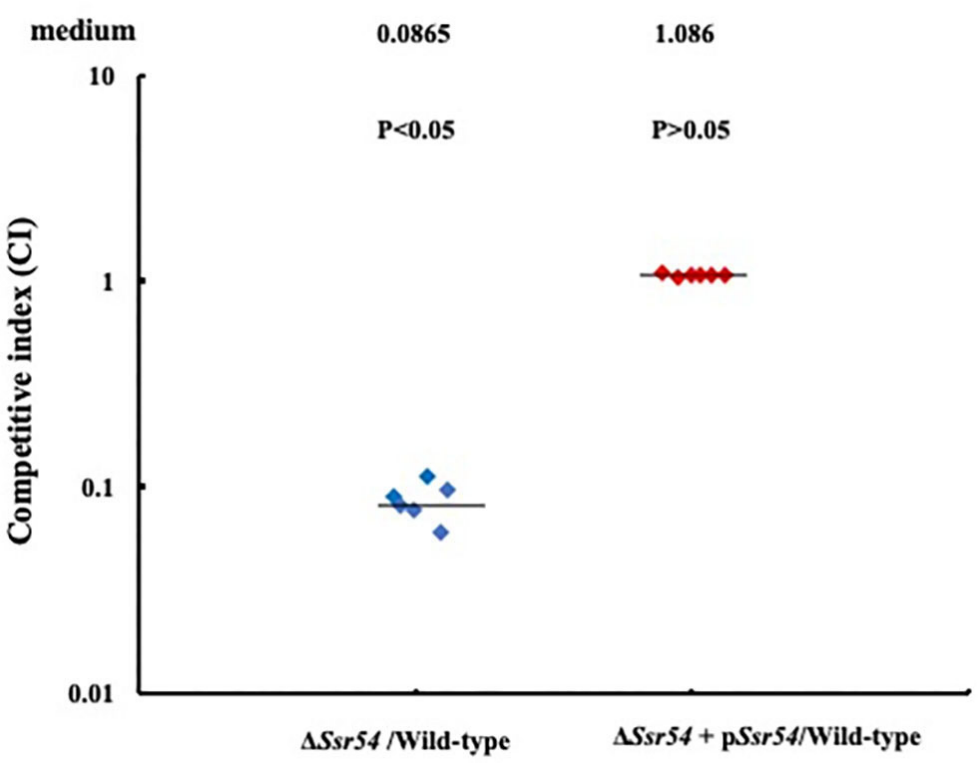
HeLa cell competitive invasion assays of wild-type, mutant Δ*Ssr54*, and complement strains. The results show the competitive index (CI) of the *Ssr54* mutant. A CI of 1 represents recovery of equivalent amounts of wild-type and mutant bacteria. The CI of the *Ssr54* mutant and wild-type was significantly lower than 1 (*P* < 0.05); The CI of the *Ssr54* complement strain and wild-type was ~1 (*P* > 0.05).

In addition, we assessed the virulence of the wild-type, *Ssr54* mutant, and complement strains in an *in vivo* HeLa cell invasion assay. The CFU recovered for the *S. flexneri* wild-type strain had a mean value of 1.696 × 10^7^ (6 replicates); the mean CFU of the wild-type was significantly higher than that of the *Ssr54* mutant (1.3 × 10^6^ CFU (6 replicates); *P* < 0.05). The calculated median value for the CI values between the *Ssr54* mutant and wild-type strains was 0.0865 (Figure 7). This value being significantly < 1 (Student's *t-*test, *P* < 0.05) strongly suggests that *Ssr54* deletion has an impact on the ability of *S. flexneri* to effectively invade epithelial cell *in vivo*. However, the total viability of the complement strain (1.7 × 10^7^) (6 replicates) was not significantly different from that of the wild-type strain (*P* > 0.05). The CI between the *Ssr54* complement and wild-type strains was 1.086 (*P* > 0.05), which was not significant (Figure 7).

### Determination of *Ssr54* Targets

To determine the targets of *Ssr54*, whole bacterial protein extracts of the wild-type and *Ssr54* mutant strains were prepared from logarithmic growth phase cultures, followed by two-dimensional PAGE. The same sample was tested in triplicate to confirm reproducibility of the two-dimensional electrophoresis maps. By comparing maps, we ascertained spots indicating highly differentially expressed proteins and conducted in-gel protease digestion, followed by mass spectroscopic analysis. Compared with the *Ssr54* mutant strain, the wild-type strain had 137 differentially expressed protein spots. Protein spots with a difference in expression of at least three-fold were identified, subjected to robotic spot excision, trypsin digested, and subsequently analyzed using MALDI-TOF mass spectrometry. Protein identification was based on highly accurate masses and MS/MS sequence data from multiple tryptic peptides. *Ssr54* deletion resulted in a total of 40 differentially expressed proteins, of which 25 were downregulated and 15 were upregulated (Supplementary Figure 2). Supplementary Figure 2A shows the wild-type strain as per our previous results (Wang L. et al., 2016); however, when compared to the *Ssr54* deletion strain, the protein spots differ. Twenty-six of the proteins differentially expressed in the wild-type and *Ssr54* mutant strains were cytoplasmic, one was periplasmic, two were on the cytoplasmic leaflet of the plasma membrane, six were on the extracellular leaflet of the plasma membrane, and four were unidentified (Table 2).

**Table 2:** Differentially expressed proteins in the small RNA mutant Δ*Ssr54 Shigella flexneri*.

**Spot no**.	**NCBI GI identifier**	**Protein description**	**pI**	**Mr**	**Protein score**	**Final_Localization**
**Downregulated protein spots in the** **Δ*****Ssr54*** **mutant**						
C04	gi|56480278	Polynucleotide phosphorylase	5.35	55089	183	Cytoplasmic
C05	gi|24114698	Glycogen branching protein	5.91	55089	216	Cytoplasmic
C06	gi|56480212	Transketolase	5.48	55089	277	Cytoplasmic
C08	gi|24111559	Pyruvate dehydrogenase dihydrolipoyltransacetylase	5.09	55089	346	Cytoplasmic
C09	gi|24114835	Glycyl-tRNAsynthetase subunit beta	5.29	55089	157	Cytoplasmic
C13	gi|24113658	BifunctionalNADH:ubiquinoneoxidoreductase subunit C/D	5.98	55089	132	Cytoplasmic
C14	gi|24112187	ABC transporter ATP-binding protein	4.99	55089	307	Cytoplasmic
C20	gi|56479850	Dihydroxyacetone kinase subunit M	4.66	55089	273	Cytoplasmic
C21	gi|24112268	Seryl-tRNAsynthetase	5.34	55089	207	Cytoplasmic
C22	gi|56480244	Outer membrane channel protein(Outer membrane channel TolC)	5.35	55089	127	Outer Membrane
C25	gi|31983549	Plasmid segregation protein (ParA)	5.66	55089	384	Cytoplasmic
C27	gi|24112337	Aromatic amino acid aminotransferase	5.54	55089	113	Cytoplasmic
C29	gi|56480132	Serine hydroxymethyltransferase	6.03	55089	76	Cytoplasmic
C33	gi|56480023	Imidazole glycerol-phosphate dehydratase/histidinol phosphatase	5.67	55089	121	Cytoplasmic
C34	gi|31983537	Plasmid stable inheritance protein	5.81	55089	246	Cytoplasmic
C35	gi|56480238	Aldehyde reductase	5.72	55089	114	Cytoplasmic
C40	gi|34491533	ftsI regulator	5.28	55089	337	Cytoplasmic
C43	gi|56479781	Outer membrane protein OmpA	5.65	55089	198	Outer Membrane
C46	gi|24112417	NAD(P)H:quinoneoxidoreductase (NAD(P)H dehydrogenase(quinone))	5.59	55089	204	Unknown
C47	gi|56479671	Adenylate kinase (AK)	5.55	55089	145	Cytoplasmic
C51	gi|56480200	Ribose-5-phosphate isomerase A	5.20	55089	96	Cytoplasmic
C52	gi|56480611	Purine nucleoside phosphorylase	5.42	55089	154	Outer Membrane
C55	gi|24112272	Hypothetical protein SF0856	5.2	55089	188	Unknown
C56	gi|56480285	PTS system sugar transporter subunit IIA	5.57	55089	128	Cytoplasmic
C62	gi|24112633	Global DNA-binding transcriptional dual regulator H-NS	5.43	55089	202	Cytoplasmic
**Upregulated protein spots in** **Δ*****Ssr54*** **mutant**						
D03	gi|24114279	Malate synthase G	5.77	55089	78	Cytoplasmic
D05	gi|24114787	Trehalase	4.91	55089	196	Cytoplasmic
D06	gi|24112385	Hydrogenase 1 large subunit	5.61	55089	346	Cytoplasmic Membrane
D08	gi|24112242	Pyruvate dehydrogenase	5.82	55089	223	Cytoplasmic Membrane
D12	gi|24114255	Outer membrane fluffing protein	5.88	55089	273	Outer Membrane
D33	gi|24113013	Adenosine deaminase	5.40	55089	208	Cytoplasmic
D35	gi|56479781	Outer membrane protein OmpA	5.65	55089	63	Outer Membrane
D36	gi|24111549	Guanosine 5'-monophosphate oxidoreductase	6.1	55089	124	Cytoplasmic
D40	gi|24112494	Hypothetical protein SF1092	4.45	55089	453	Cytoplasmic
D41	gi|24113371	DgsA anti-repressor MtfA	4.63	55089	232	Unknown
D44	gi|24113539	Methyl-galactoside ABC transporter substrate-binding protein MglB(GBP)	5.68	55089	242	Periplasmic
D51	gi|56479997	Trehalose-6-phosphate phosphatase	5.38	55089	258	Unknown
D54	gi|56479781	Outer membrane protein OmpA	5.65	55089	152	Outer Membrane
D57	gi|56479781	Outer membrane protein OmpA	5.65	55089	449	Outer Membrane
D69	gi|24112387	Hydrogenase 1 maturation protease	4.81	55089	64	Cytoplasmic

*pI represents the isoelectric point of the protein sample; Mr represents the molecular mass of the protein sample*.

After *Ssr54* deletion, mRNA of proteins with the most significantly altered expression levels (TreF, OmpA [C43], and TolC) and their roles in stress tolerance and virulence were analyzed by qRT-PCR. The *tolC* and *ompA* genes were significantly downregulated in the *Ssr54* mutant strain compared to those in the wild-type strain, whereas *treF* was significantly upregulated in the *Ssr54* mutant strain.

**Figure 8:**
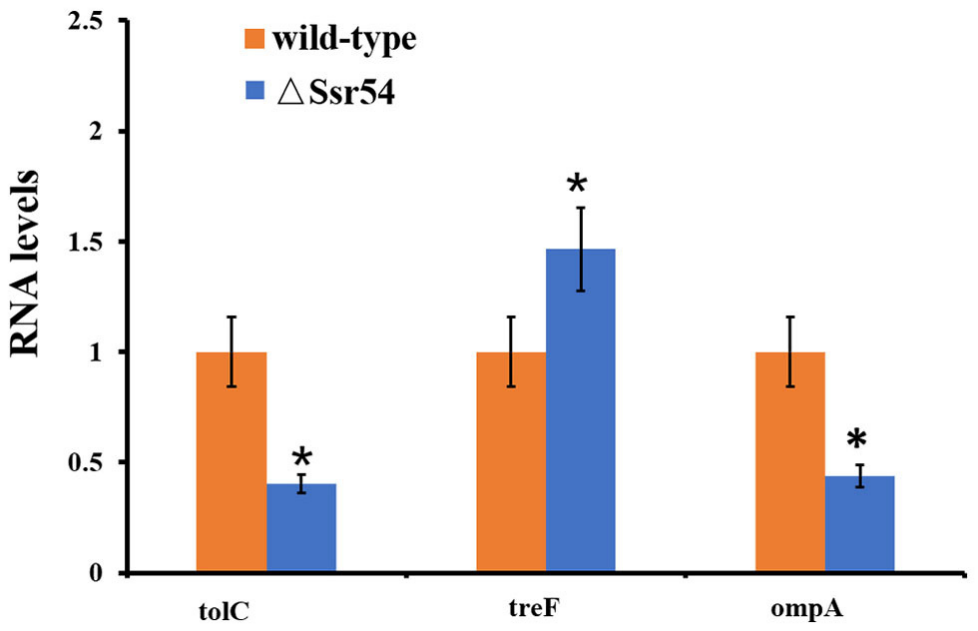
qRT-PCR for three mRNA targets of *Ssr54*. Level of three mRNA targets in the Δ*Ssr54* mutant strain under hyperosmotic conditions expressed relative to the level in wild-type. Statistical analyses were performed using Student's *t*-test. Data are representative of three independent experiments. Error bars indicate one standard deviation. *Denotes a significant difference with *P* < 0.01.

To determine whether hyperosmotic conditions affect the expression of these target genes, *treF, tolC*, and *ompA* expression levels were determined under hyperosmotic conditions using qRT-PCR (Figure 8). Hyperosmotic conditions activated *treF* expression (1.99-fold) (*P* = 0.0115) and downregulated *tolC* and *ompA* expression (approximately two-fold) (*P* = 0.0207 and 0.0218, respectively).

## Discussion

Numerous recent studies have investigated bacterial sRNAs, with more than 900 novel sRNAs reported in the sRNA database RNAMap (Huang et al., 2009). Of these, 79 were found in *E. coli*. However, there have been very few functional studies of sRNAs, and only 57 sRNA targets have been identified as per the sRNATarBase database (Wang L. et al., 2016). Hence, investigations on the mechanism of action of novel sRNAs are warranted. Interestingly, the sequence of sRNA *ArrS*, identified in *E. coli* strain K-12, is identical to that of *Ssr54* (Bianco et al., 2019). However, there are significant differences between their lengths and functions. The length of *ArrS* is 50 nt, which is less than that of *Ssr54*. *ArrS* regulates the response of *E. coli* to low pH, whereas *Ssr54* demonstrates different functions in *S. flexneri*. In the present study, we confirmed the presence of a novel sRNA, *Ssr54*, in *S. flexneri*, observed its role in environmental stress tolerance and virulence, and identified possible targets to suggest mechanisms of action.

In pathogenic bacteria, response to osmotic stress is a critical mechanism for survival during environmental osmotic stress as well as for establishing infection. For instance, the ProU system functions to allow *S. sonnei* to tolerate hyperosmotic stress *in vitro*, while also facilitating the survival and proliferation of the bacteria within stressful intracellular niches (Mahmoud et al., 2017). Herein, we observed that when exposed to hyperosmotic stress, *Ssr54* was downregulated and survival of the *Ssr54* deletion strain increased. Generally, bacteria tolerate changes in osmolarity by solute efflux or gene regulation (Mahmoud et al., 2017), allowing them to adapt for survival. In this way, the downregulation of *Ssr54* may serve as a protective mechanism for *S. flexneri* in the gastrointestinal tract. Furthermore, *Ssr54* deletion was shown to upregulate expression of the target gene *treF*. TreF, the cytosolic trehalase, is weakly induced by high osmolarity of the medium and is partially dependent on RpoS, the stationary - phase sigma factor. Hence, *treF* is expressed primarily under hyperosmotic conditions (Horlacher et al., 1996; Weber et al., 2006); accordingly, *Ssr54* expression seems to inhibit *treF*, with *treF* upregulated under high osmotic concentrations when *Ssr54* is repressed. *Ssr54* may, therefore, upregulate *treF* to enhance survival under osmotic stress. Thus, virulence genes in Shigella are expressed in response to increases in osmolarity.

*Shigella* guinea pig Sereny tests, lung invasion assays, and HeLa invasion assays were also performed to determine whether *Ssr54* regulates *S. flexneri* virulence. Results suggest that the virulence of *S. flexneri* Δ*Ssr54* was significantly decreased as compared to that of the wild-type strain. TolC is an outer membrane channel protein that contributes to bacterial adhesion, antibiotic resistance, and invasion during disease pathogenesis (Stone and Miller, 1995; Sharff et al., 2001; Platz et al., 2010). *Ssr54* deletion downregulated *tolC*, which may have attenuated the virulence of *S. flexneri*. Our study also showed that deletion of *Ssr54* downregulated *ompA*. OmpA, the major outer membrane protein, plays important roles in the virulence of pathogens (Scribano et al., 2016). Some studies have suggested that *micA* and *rseX* sRNAs bind to the *ompA* translation initiation region and block ribosome binding to prevent expression of *ompA* in *E. coli* (Udekwu et al., 2005). Hence, we speculate that *ompA* was downregulated in the *Ssr54* deletion strain, decreasing the virulence of *S. flexneri*. Overall, *Ssr54* may affect *S. flexneri* virulence by regulating the expression of putative target genes *tolC* and *ompA*, either directly or indirectly.

Studies have suggested that hyperosmotic pressures affect virulence gene expression in *S. flexneri* (Porter and Dorman, 1994). Under hyperosmotic stress, the hyperosmotic regulator gene *treF* was upregulated, whereas the virulence regulator genes *tolc* and *ompA* were downregulated, indicating that *Ssr54* regulates *treF* to increase *S. flexneri* tolerance to hyperosmotic conditions and regulates virulence genes *tolc* and *ompA* to reduce pathogenicity of this bacterium. These results have, therefore, established the role of *Ssr54* in survival of *Shigella* under hyperosmotic environments; however, whether it is required in the context of infection remains unknown. Moreover, whether *Ssr54* regulates virulence and hyperosmotic stress tolerance by regulating genes directly, or via other pathways, remains to be answered; we are currently investigating this mechanism.

## Data Availability Statement

The raw/processed data required to reproduce these findings cannot be shared at this time as the data also forms part of an ongoing study.

## Ethics Statement

The animal study was reviewed and approved by the Ethics Committee for Animal Experimentation of the Academy of Military Medical Sciences.

## Author Contributions

HS, LW, and HM designed the research, assessed and interpreted the results, and prepared the manuscript. GY, BL, LJ, HQ, MY, and BZ carried out the data analysis and designed the experiments. JX, SQ, and PL assisted in carrying out the experiments. All authors contributed to the article and approved the submitted version.

## Conflict of Interest

The authors declare that the research was conducted in the absence of any commercial or financial relationships that could be construed as a potential conflict of interest.

## References

[B1] Bianco C. M. Fröhlich K. S. Vanderpool C. K. (2019). Bacterial cyclopropane fatty acid synthase mRNA is targeted by activating and repressing small RNAs. J. Bacteriol. 201:e00461-19. 10.1128/JB.00461-19 31308070 PMC6755755

[B2] Bobrovskyy M. Vanderpool C. K. (2013). Regulation of bacterial metabolism by small RNAs using diverse mechanisms. Annu. Rev. Genet. 47, 209–232. 10.1146/annurev-genet-111212-133445 24016191

[B3] Christiansen J. K. Larsen M. H. Ingmer H. Søgaard-Andersen L. Kallipolitis B. H. (2004). The RNA-binding protein Hfq of *Listeria monocytogenes*: role in stress tolerance and virulence. J. Bacteriol. 186, 3355–3362. 10.1128/JB.186.11.3355-3362.2004 15150220 PMC415768

[B4] De Lay N. Schu D. J. Gottesman S. (2013). Bacterial small RNA-based negative regulation: Hfq and its accomplices. J. Biol. Chem. 288, 7996–8003. 10.1074/jbc.R112.441386 23362267 PMC3605619

[B5] Fris M. E. Broach W. H. Klim S. E. Coschigano P. W. Carroll R. K. Caswell C. C.. (2017). Sibling sRNA RyfA1 influences *Shigella* dysenteriae pathogenesis. Genes 8:50. 10.3390/genes8020050 28134784 PMC5333039

[B6] Fris M. E. Murphy E. R. (2016). Riboregulators: fine-tuning virulence in *Shigella*. Front. Cell Infect. Microbiol. 6:2. 10.3389/fcimb.2016.00002 26858941 PMC4728522

[B7] Giangrossi M. Giuliodori A. M. Tran C. N. Amici A. Marchini C. Falconi M. (2017). VirF relieves the transcriptional attenuation of the virulence gene icsA of *Shigella flexneri* affecting the icsA mRNA-RnaG complex formation. Front. Microbiol. 8:650. 10.3389/fmicb.2017.00650 28458662 PMC5394118

[B8] Giangrossi M. Prosseda G. Tran C. N. Brandi A. Colonna B. Falconi M. (2010). A novel antisense RNA regulates at transcriptional level the virulence gene icsA of *Shigella flexneri*. Nucleic Acids Res. 38, 3362–3375. 10.1093/nar/gkq025 20129941 PMC2879508

[B9] Gong H. Vu G. P. Bai Y. Chan E. Wu R. Yang E.. (2011). A *Salmonella* small non-coding RNA facilitates bacterial invasion and intracellular replication by modulating the expression of virulence factors. PLoS Pathog. 7:e1002120. 10.1371/journal.ppat.1002120 21949647 PMC3174252

[B10] Gottesman S. Storz G. (2011). Bacterial small RNA regulators: versatile roles and rapidly evolving variations. Cold Spring Harb. Perspect. Biol. 3:a003798. 10.1101/cshperspect.a003798 20980440 PMC3225950

[B11] Horlacher R. Uhland K. Klein W. Ehrmann M. Boos W. (1996). Characterization of a cytoplasmic trehalase of *Escherichia coli*. J. Bacteriol. 178, 6250–6257. 10.1128/JB.178.21.6250-6257.1996 8892826 PMC178497

[B12] Huang H. Y. Chang H. Y. Chou C. H. Tseng C. P. Ho S. Y. Yang C. D.. (2009). sRNAMap: genomic maps for small non-coding RNAs, their regulators and their targets in microbial genomes. Nucleic Acids Res. 37, D150–D154. 10.1093/nar/gkn852 19015153 PMC2686527

[B13] Kotloff K. L. Winickoff J. P. Ivanoff B. Clemens J. D. Swerdlow D. L. Sansonetti P. J.. (1999). Global burden of *Shigella* infections: implications for vaccine development and implementation of control strategies. Bull. World Health Organ. 77, 651–666. 10516787 PMC2557719

[B14] Lenz D. H. Mok K. C. Lilley B. N. Kulkarni R. V. Wingreen N. S. Bassler B. L. (2004). The small RNA chaperone Hfq and multiple small RNAs control quorum sensing in *Vibrio harveyi* and *Vibrio cholerae*. Cell 118, 69–82. 10.1016/j.cell.2004.06.009 15242645

[B15] Leuzzi A. Di Martino M. L. Campilongo R. Falconi M. Barbagallo M. Marcocci L.. (2015). Multifactor regulation of the MdtJI polyamine transporter in *Shigella*. PLoS ONE 10:e0136744. 10.1371/journal.pone.0136744 26313003 PMC4636849

[B16] Lewis B. P. Burge C. B. Bartel D. P. (2005). Conserved seed pairing, often flanked by adenosines, indicates that thousands of human genes are microRNA targets. Cell 120, 15–20. 10.1016/j.cell.2004.12.035 15652477

[B17] Mahmoud R. Y. Li W. Eldomany R. A. Emara M. Yu J. (2017). The *Shigella* ProU system is required for osmotic tolerance and virulence. Virulence 8, 362–374. 10.1080/21505594.2016.1227906 27558288 PMC5477723

[B18] Mangold M. Siller M. Roppenser B. Vlaminckx B. J. Penfound T. A. Klein R.. (2004). Synthesis of group A streptococcal virulence factors is controlled by a regulatory RNA molecule. Mol. Microbiol. 53, 1515–1527. 10.1111/j.1365-2958.2004.04222.x 15387826

[B19] Massé E. Gottesman S. (2002). A small RNA regulates the expression of genes involved in iron metabolism in *Escherichia coli*. Proc. Natl. Acad. Sci. U.S.A. 99, 4620–4625. 10.1073/pnas.032066599 11917098 PMC123697

[B20] Massé E. Vanderpool C. K. Gottesman S. (2005). Effect of RyhB small RNA on global iron use in *Escherichia coli*. J. Bacteriol. 187, 6962–6971. 10.1128/JB.187.20.6962-6971.2005 16199566 PMC1251601

[B21] Michaux C. Hartke A. Martini C. Reiss S. Albrecht D. Budin-Verneuil A.. (2014). Involvement of *Enterococcus faecalis* small RNAs in stress response and virulence. Infect. Immun. 82, 3599–3611. 10.1128/IAI.01900-14 24914223 PMC4187846

[B22] Murina V. N. Nikulin A. D. (2015). Bacterial small regulatory RNAs and Hfq protein. Biochemistry 80, 1647–1654. 10.1134/S0006297915130027 26878571

[B23] Murphy E. R. Payne S. M. (2007). RyhB, an iron-responsive small RNA molecule, regulates *Shigella dysenteriae* virulence. Infect. Immun. 75, 3470–3477. 10.1128/IAI.00112-07 17438026 PMC1932958

[B24] Nickerson K. P. Chanin R. B. Sistrunk J. R. Rasko D. A. Fink P. J. Barry E. M.. (2017). Analysis of *Shigella flexneri* resistance, biofilm formation, and transcriptional profile in response to bile salts. Infect. Immun. 85, e01067–e01016. 10.1128/IAI.01067-16 28348056 PMC5442615

[B25] Park S. H. Oh K. H. Kim C. K. (2001). Adaptive and cross-protective responses of *Pseudomonas* sp. DJ-12 to several aromatics and other stress shocks. Curr. Microbiol. 43, 176–181. 10.1007/s002840010283 11400066

[B26] Pichon C. Felden B. (2005). Small RNA genes expressed from *Staphylococcus aureus* genomic and pathogenicity islands with specific expression among pathogenic strains. Proc. Natl. Acad. Sci. U.S.A. 102, 14249–14254. 10.1073/pnas.0503838102 16183745 PMC1242290

[B27] Platz G. J. Bublitz D. C. Mena P. Benach J. L. Furie M. B. Thanassi D. G. (2010). A tolC mutant of *Francisella tularensis* is hypercytotoxic compared to the wild type and elicits increased proinflammatory responses from host cells. Infect. Immun. 78, 1022–1031. 10.1128/IAI.00992-09 20028804 PMC2825903

[B28] Porter M. E. Dorman C. J. (1994). A role for H-NS in the thermo-osmotic regulation of virulence gene expression in *Shigella flexneri*. J. Bacteriol. 176, 4187–4191. 10.1128/JB.176.13.4187-4191.1994 8021202 PMC205622

[B29] Scribano D. Damico R. Ambrosi C. Superti F. Marazzato M. Conte M. P.. (2016). The *Shigella flexneri* OmpA amino acid residues 188 EVQ 190 are essential for the interaction with the virulence factor PhoN2. Biochem. Biophys. Rep. 8, 168–173. 10.1016/j.bbrep.2016.08.010 28955953 PMC5613738

[B30] Sereny B. (1955). Experimental *Shigella* keratoconjunctivitis; a preliminary report. Acta. Microbiol. 2, 293–296. 14387586

[B31] Sharff A. Fanutti C. Shi J. Calladine C. Luisi B. (2001). The role of the TolC family in protein transport and multidrug efflux. From stereochemical certainty to mechanistic hypothesis. Eur. J. Biochem. 268, 5011–5026. 10.1046/j.0014-2956.2001.02442.x 11589692

[B32] Small P. Blankenhorn D. Welty D. Zinser E. Slonczewski J. L. (1994). Acid and base resistance in *Escherichia coli* and *Shigella flexneri*: role of rpoS and growth pH. J. Bacteriol. 176, 1729–1737. 10.1128/JB.176.6.1729-1737.1994 8132468 PMC205261

[B33] Stone B. J. Miller V. L. (1995). *Salmonella enteritidis* has a homologue of tolC that is required for virulence in BALB/c mice. Mol. Microbiol. 17, 701–712. 10.1111/j.1365-2958.1995.mmi_17040701.x 8801424

[B34] Udekwu K. I. Darfeuille F. Vogel J. Reimegård J. Holmqvist E. Wagner E. G. (2005). Hfq-dependent regulation of OmpA synthesis is mediated by an antisense RNA. Genes Dev. 19, 2355–2366. 10.1101/gad.354405 16204185 PMC1240044

[B35] Vanderpool C. K. Balasubramanian D. Lloyd C. R. (2011). Dual-function RNA regulators in bacteria. Biochimie 93, 1943–1949. 10.1016/j.biochi.2011.07.016 21816203 PMC3185123

[B36] Wagner E. G. H. Romby P. (2015). Small RNAs in bacteria and archaea: who they are, what they do, and how they do it. Adv. Genet. 90, 133–208. 10.1016/bs.adgen.2015.05.001 26296935

[B38] Wang L. Yang G. Qi L. Li X. Jia L. Xie J.. (2016). A novel small RNA regulates tolerance and virulence in *Shigella flexneri* by responding to acidic environmental changes. Front. Cell. Infect. Microbiol. 6:24. 10.3389/fcimb.2016.00024 27014636 PMC4782007

[B39] Wang X. Y. Tao F. Xiao D. Lee H. Deen J. Gong J.. (2006). Trend and disease burden of bacillary dysentery in China (1991–2000). Bull. World Health Organ. 84, 561–568. 10.2471/BLT.05.023853 16878230 PMC2627389

[B40] Wang Y. Bai Y. Qu Q. Xu J. Chen Y. Zhong Z.. (2011). The 16MΔvjbR as an ideal live attenuated vaccine candidate for differentiation between *Brucella* vaccination and infection. Vet. Microbiol. 151, 354–362. 10.1016/j.vetmic.2011.03.031 21530111

[B41] Weber A. Kögl S. A. Jung K. (2006). Time-dependent proteome alterations under osmotic stress during aerobic and anaerobic growth in *Escherichia coli*. J. Bacteriol. 188, 7165–7175. 10.1128/JB.00508-06 17015655 PMC1636219

