# Supplementary material for: A Novel sRNA in Shigella flexneri That Regulates Tolerance and Virulence Under Hyperosmotic Pressure
Source: Front Cell Infect Microbiol. 2020 Sep 16;10:483. doi: 10.3389/fcimb.2020.00483 (PMC7526569; doi:10.3389/fcimb.2020.00483)
Supplement: Supplementary file 1 [file Data_Sheet_1.docx]

Supplementary Material

# Supplementary Figures and Tables

## Supplementary Figures


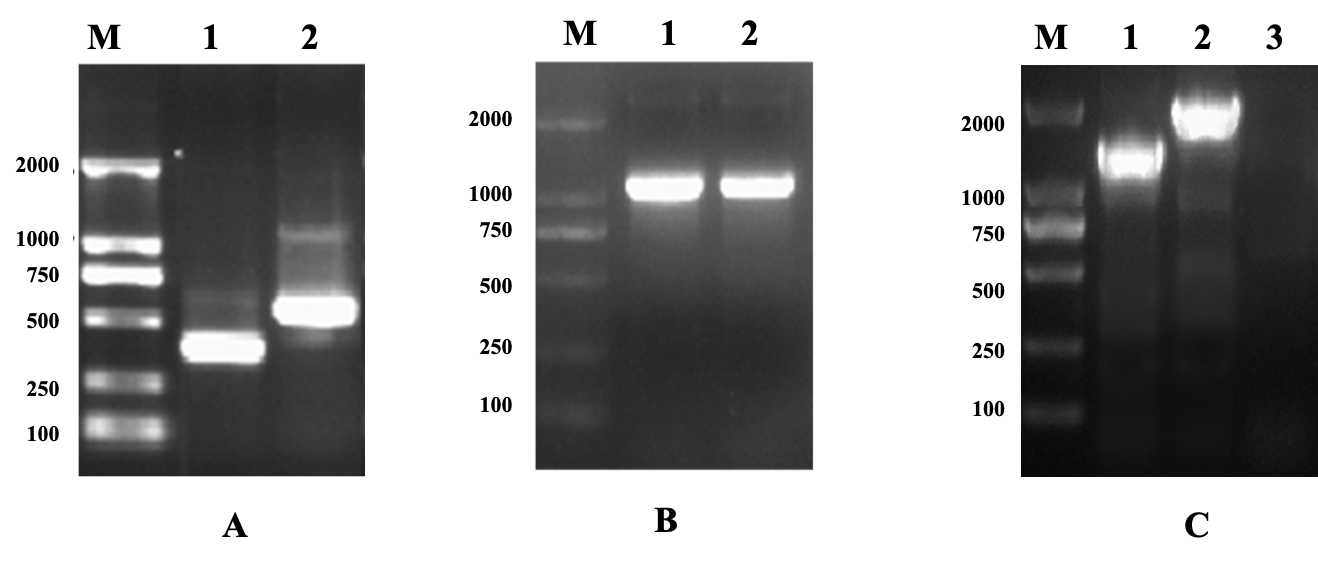


**Supplementary Figure 1.** **Verification for construction of the *Ssr54* mutant of *S. flexneri.*** (**A**) M, marker; 1, upstream of sRNA (*Ssr54*); 2, downstream of sRNA (*Ssr54*) in *S. flexneri* wild-type, *Ssr54* mutant, and *Ssr54* complement strains. *Ssr54* expression in the *S. flexneri* strains grown to logarithmic growth phase was verified by Northern blotting; (**B**) M, marker; 1, 2, the kana gene (**C**) M, marker; 1, the kana interior verification primer in *S. flexneri* wild-type; 2, the kana external verification primer in *S. flexneri* wild-type; 3, sRNA (*Ssr54*) primer in *S. flexneri* wild-type.


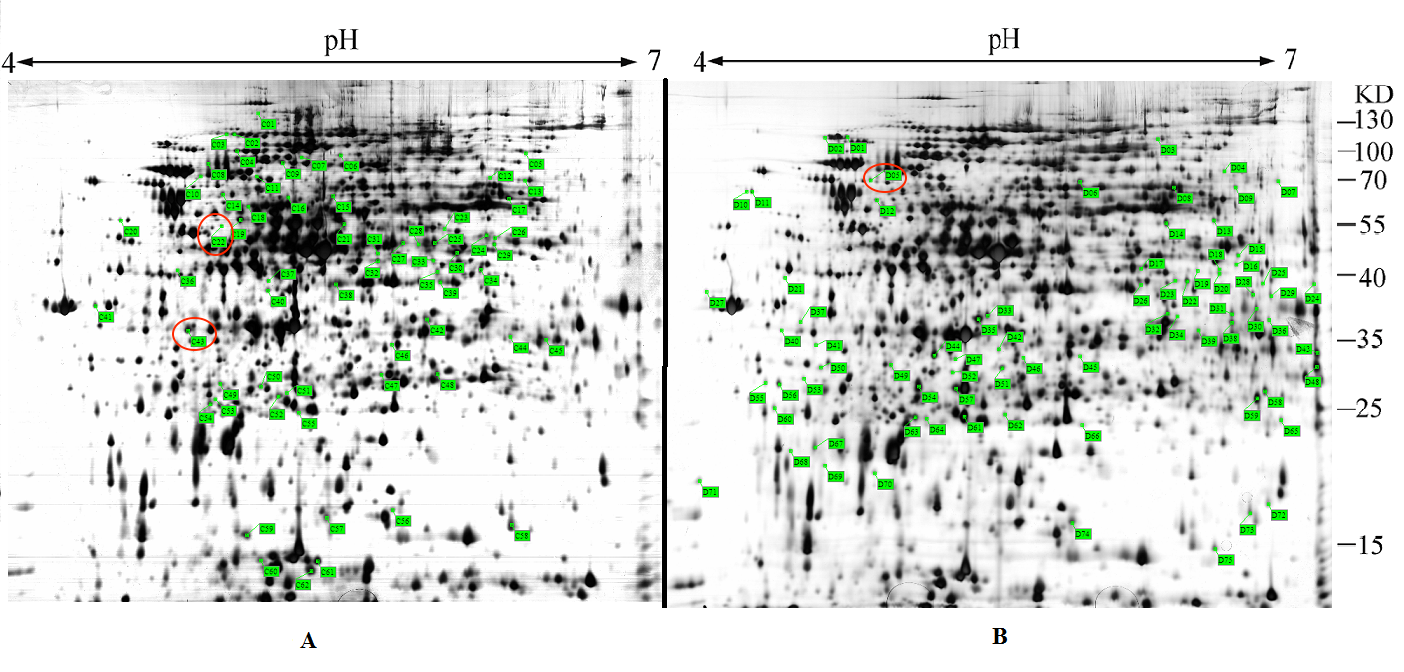


**Supplementary Figure 2.** **Representative two-dimensional polyacrylamide gel electrophoresis of *S. flexneri* wild-type 301 and *ΔSsr54* strains.** Protein expression levels in the wild-type (**A**) and *ΔSsr54* mutant (**B**) in the stationary phase. The highlighted spots in (A) represent proteins that have greater expression levels in the wild-type, whereas those in (B) represent proteins that are more abundant in the *ΔSsr54* strain. The three genes of TreF(D05), OmpA(C43), and TolC(C22) spots were circled in red.


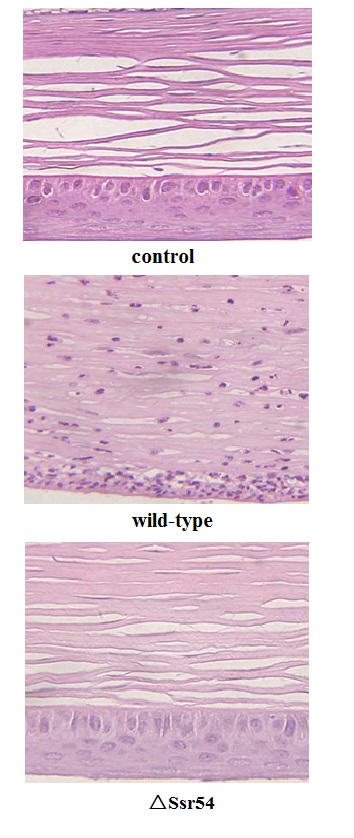


**Supplementary Figure 3.** **Corneal pathology of wild-type *S flexneri* and the sRNA mutant Δ*Ssr54*.** More inflammatory cells were observed in the wild-type strain than in the mutant Δ*Ssr54* strain, whereas the NaCl control group had no inflammatory cells. (**A**) control, (**B**) wild-type, and (**C**) Δ*Ssr54* strain.
